# Supplementary material for: A mesocortical glutamatergic pathway modulates neuropathic pain independent of dopamine co-release
Source: Nat Commun. 2024 Jan 20;15:643. doi: 10.1038/s41467-024-45035-2 (PMC10799877; doi:10.1038/s41467-024-45035-2)
Supplement: Supplementary file 1 — Supplementary Information [file 41467_2024_45035_MOESM1_ESM.pdf]

Supplementary Information

A mesocortical glutamatergic pathway modulates neuropathic pain independent of dopamine co-release

Miao Li[1] & Guang Yang[1*]

[1] Department of Anesthesiology, Columbia University Irving Medical Center, New York, New York, 10032, USA

* Correspondence: gy2268@cumc.columbia.edu

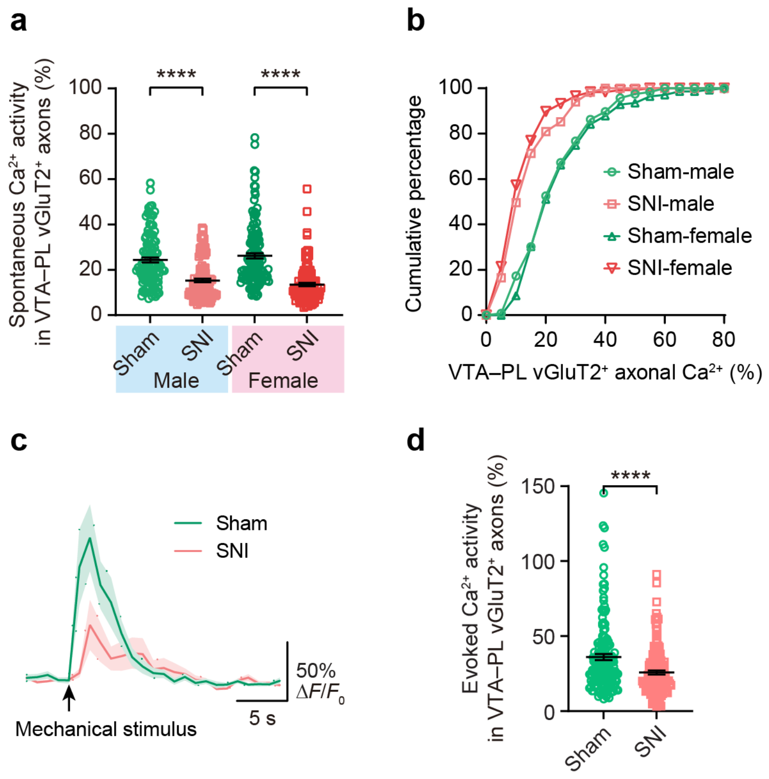

Supplementary Fig. 1: VTA–PL glutamatergic activity is decreased in both male and female mice with neuropathic pain.

a, SNI reduces spontaneous Ca^{2+} activity in VTA–PL glutamatergic axons in both males ($P <$ 0.0001; n = 116, 115 axon segments from three mice per group) and females ($P < 0.0001$; n = 139, 117 axon segments from three mice per group). There is no significant difference between the sexes (sham, $P = 0.4516$; SNI, $P = 0.1345$). **b**, Distribution plot of data shown in **a**. **c**, Population average response of VTA glutamatergic terminals in the PL before and after mechanical stimulation. Shading indicates S.E.M. **d**, Sensory stimulus-evoked Ca^{2+} activity in VTA–PL glutamatergic terminals is lower in SNI mice than sham mice ($P < 0.0001$; n = 172 and 145 axon segments from six mice per group). Each dot indicates data from a single axon segment (**a**, **d**). Summary data are presented as mean ± S.E.M. ****$P < 0.0001$; by two-sided Mann-Whitney test (**a**, **d**). Source data are provided as a Source Data file.

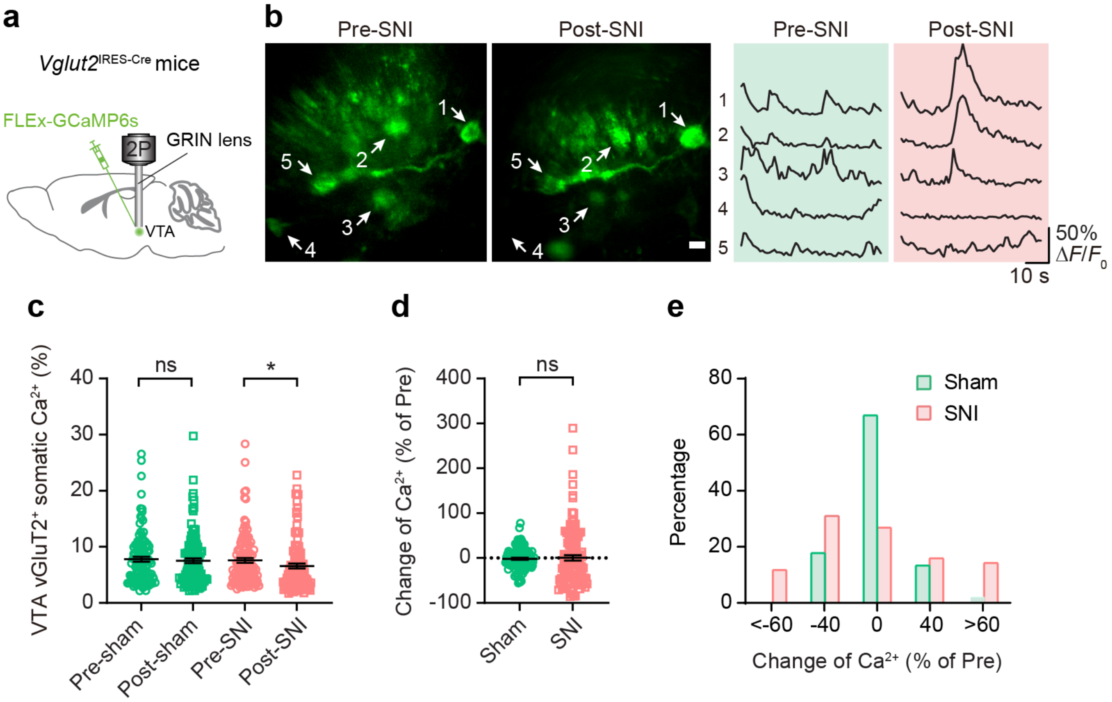

Supplementary Fig. 2: Analysis of the somatic activity of VTA glutamatergic neurons in mice with neuropathic pain.

a, Experimental design for expressing GCaMP6 in VTA glutamatergic neurons and *in vivo* two-photon (2P) Ca^{2+} imaging in the VTA through a GRIN lens. **b**, Representative two-photon images of VTA glutamatergic neurons expressing GCaMP6s (left) and their corresponding Ca^{2+} traces before and two weeks after SNI (right). Scale bar, 20 μm. **c**, Somatic Ca^{2+} activity before and two weeks after sham or SNI surgery (sham, $P = 0.1636$, $n = 112$ cells from ten mice; SNI, $P = 0.0103$, $n = 119$ cells from nine mice). **d**, Changes in somatic Ca^{2+} activity after sham or SNI surgery ($P = 0.0998$). **e**, Distribution of changes in somatic Ca^{2+} activity after sham or SNI surgery. The distribution of SNI-induced changes in neuronal activity appears to be broader compared to the sham group, suggesting that VTA glutamatergic neurons may exhibit distinct responses to neuropathic pain based on their specific circuits. Summary data are presented as mean ± S.E.M. *$P < 0.05$; ns, not significant; by Wilcoxon test (**c**) or Mann-Whitney test (**d**), two-sided. Source data are provided as a Source Data file.

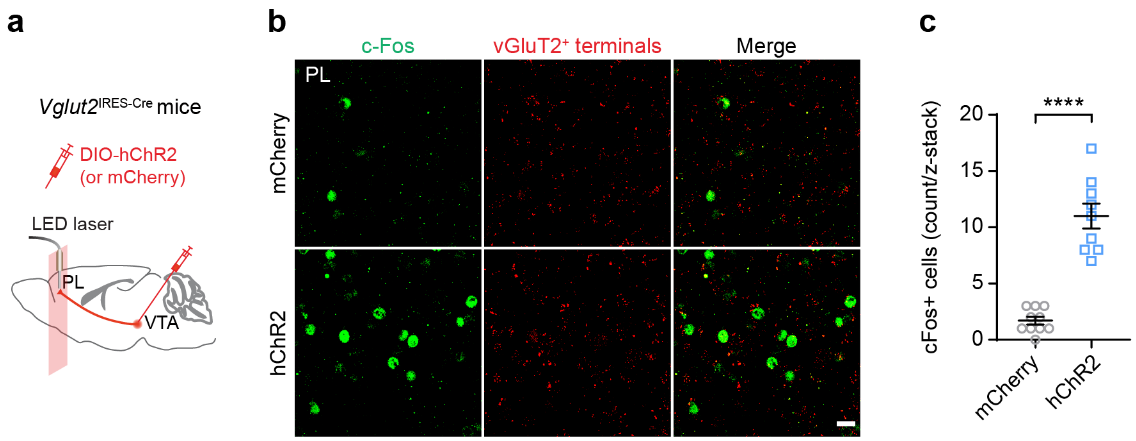

Supplementary Fig. 3: Optogenetic stimulation of VTA–PL glutamatergic terminals activates PL.

a, Experimental design for expressing hChR2 or mCherry (control) in VTA glutamatergic (vGluT2^{+}) neurons and stimulating their terminals in the PL. **b**, Representative images of PL immunostained for c-Fos, a neuronal activity marker. **c**, Optical stimulation of VTA–PL glutamatergic terminals expressing hChR2 results in increased c-Fos expression in the PL ($P <$ 0.0001, n = 10, 9 slices from three mice per group). Summary data are presented as mean ± S.E.M. ****$P < 0.0001$; by two-sided Mann-Whitney test. Source data are provided as a Source Data file.

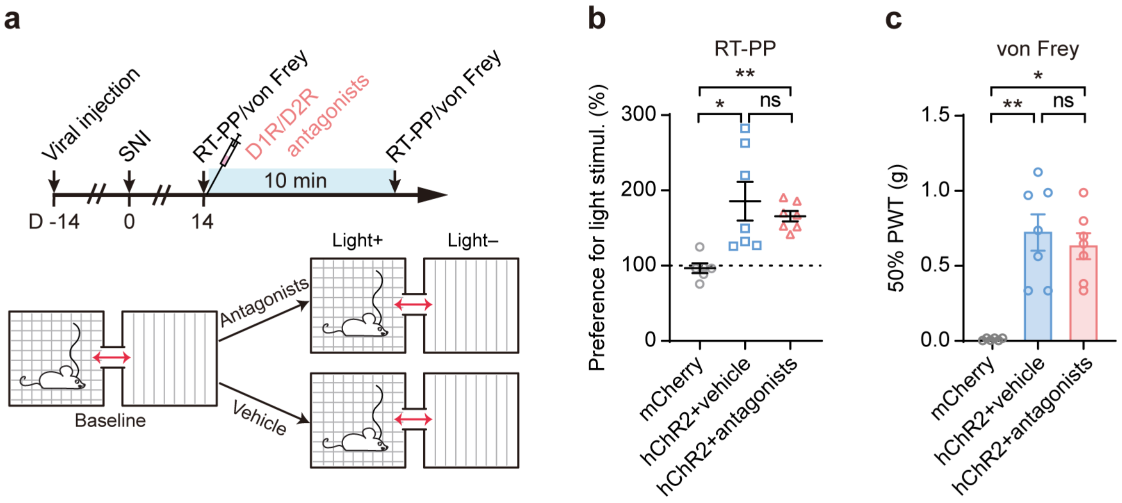

Supplementary Fig. 4: VTA–PL glutamatergic pathway modulates pain aversion and touch hypersensitivity absent of dopamine.

a, Top, experimental timeline for viral injection, SNI, and behavior tests before and after administration of D1R/D2R antagonists (SCH23390 and sulpiride) or vehicle. Bottom, schematic showing real-time place preference (RT-PP) tests in SNI mice treated with antagonists or vehicle. **b**, Activating VTA–PL glutamatergic terminals induces place preference in mice administered with antagonists ($P = 0.0050$) or vehicle ($P = 0.0102$). There is no difference between antagonists and vehicle-treated groups ($P > 0.9999$). **c**, Activating VTA–PL glutamatergic terminals increases mechanical thresholds in mice administered with antagonists ($P = 0.0105$) or vehicle ($P = 0.0044$) ($n = 6, 7, 7$ mice). There is no difference between antagonists and vehicle-treated groups ($P > 0.9999$). Mice in the mCherry group are the same mice shown in Fig. 2. Each dot indicates data from a single animal. Summary data are presented as mean ± S.E.M. *$P < 0.05$, **$P < 0.01$; ns, not significant; by Kruskal-Wallis test followed by Dunn's multiple comparisons test. Source data are provided as a Source Data file.

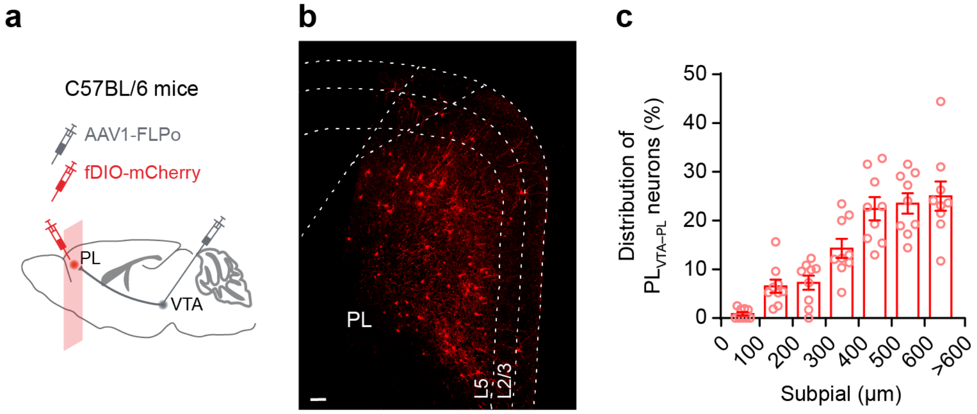

Supplementary Fig. 5: Distribution of PL neurons receiving VTA inputs.

a, Experimental design for expressing mCherry in PL neurons receiving VTA projections ($PL_{VTA–PL}$ neurons). **b**, Representative confocal image showing mCherry-labelled $PL_{VTA–PL}$ neurons. Scale, 100 µm. **c**, Distribution of $mCherry^+$ somas across layers of PL ($n = 9$ slices from four mice). Source data are provided as a Source Data file.

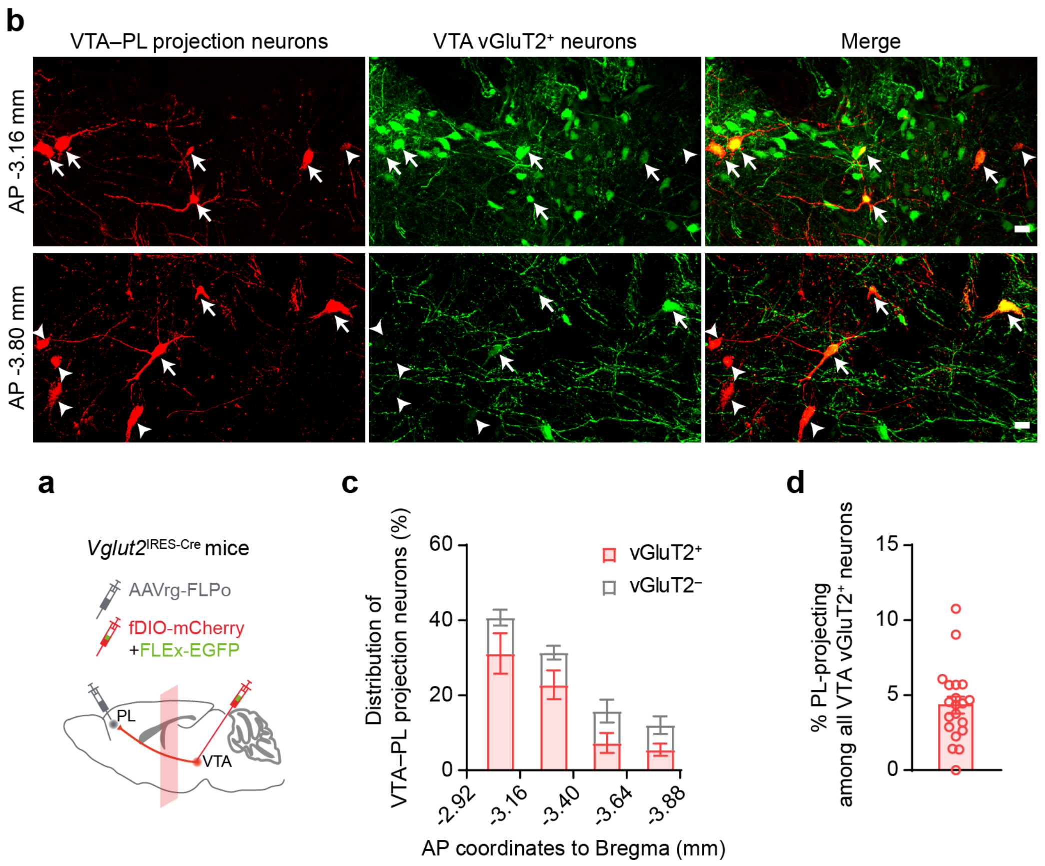

Supplementary Fig. 6: The majority of neurons projecting to the PL in the anterior VTA are glutamatergic.

a, Experimental design for expressing mCherry in VTA neurons projecting to PL (VTA–PL projection neurons) and EGFP in VTA glutamatergic (vGluT2^{+}) neurons. **b**, Confocal images of VTA showing colocalization of mCherry-labeled VTA–PL projection neurons and EGFP-labeled VTA vGluT2^{+} neurons. Arrows indicate VTA–PL vGluT2^{+} neurons; Arrowheads indicate VTA–PL vGluT2^{-} neurons. Scale bar, 20 µm. **c**, In the anterior VTA (AP -2.92 ~ -3.40 mm), most of the PL-projecting neurons are glutamatergic (*n* = 8 mice). **d**, Percentage of PL-projecting glutamatergic neurons among all glutamatergic neurons in the VTA (*n* = 20 slices from four mice). Summary data are presented as mean ± S.E.M. Source data are provided as a Source Data file.

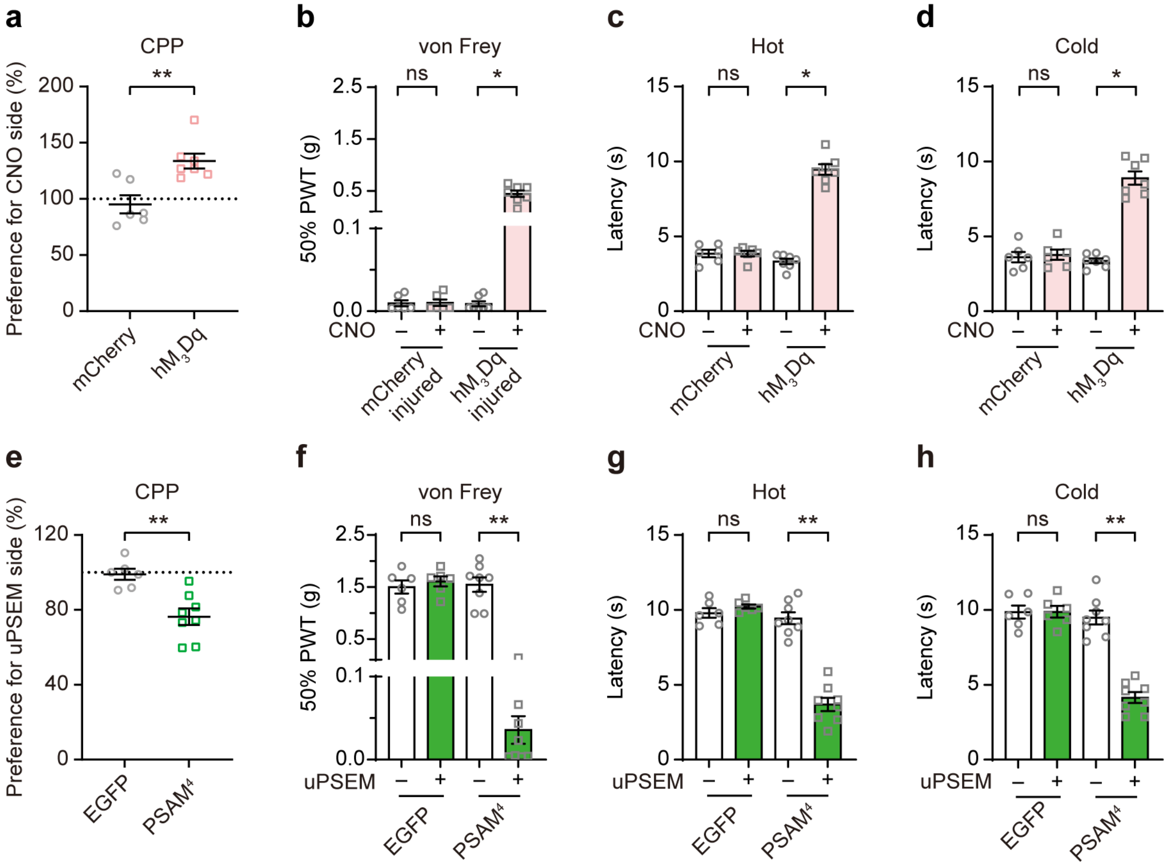

Supplementary Fig. 7: Chemogenetic manipulation of VTA–PL projections regulates pain-associated behaviors.

a, Preference to stay in CNO-paired chamber for SNI mice expressing mCherry or hM_3Dq in contralateral VTA–PL projection neurons (*P* = 0.0047). **b**–**d**, Measurements of nociceptive thresholds in SNI mice expressing mCherry or hM_3Dq. Activation of VTA–PL projection neurons increases the animals' mechanical (*P* = 0.0156), hot (*P* = 0.0156), and cold (*P* = 0.0156) thresholds in the limb ipsilateral to SNI. CNO injection has no effect on mechanical and thermal sensitivity in SNI mice expressing mCherry. In **a**–**d**, *n* = 6, 7 mice for mCherry, hM_3Dq respectively. **e**, Preference to stay in uPSEM817-paired chamber for naïve mice expressing EGFP or $PSAM^4$-GlyR in VTA–PL projection neurons (*P* = 0.0027). **f**–**h**, Inhibition of VTA–PL projections reduces the animal's mechanical (*P* = 0.0078), hot (*P* = 0.0078), and cold (*P* = 0.0078) thresholds in the limb contralateral to the viral injection side. Applying uPSEM817 has no effect on mechanical and thermal sensitivity in mice expressing EGFP. In **e**–**h**, *n* = 6, 8 mice for EGFP, $PSAM^4$-GlyR respectively. Each dot indicates data from a single animal. Summary data are presented as mean ± S.E.M. $^*P < 0.05$, $^{**}P < 0.01$; ns, not significant; by Mann-Whitney test for unpaired comparison or Wilcoxon test for paired comparison, two-sided. Source data are provided as a Source Data file.

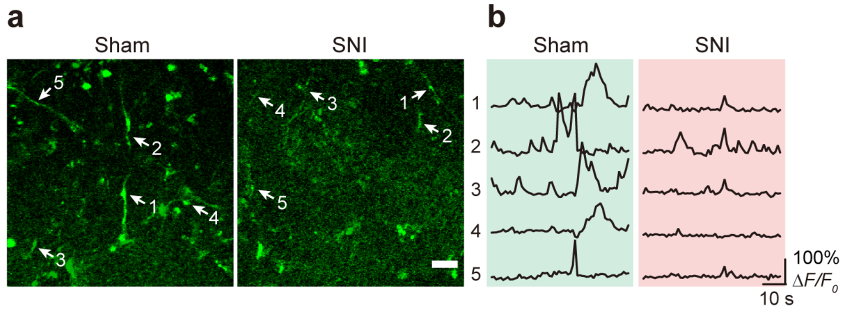

Supplementary Fig. 8: *In vivo* Ca^{2+} imaging of $PL_{VTA–PL}$ axonal terminals in the ACC.

Representative Ca^{2+} images (**a**) and fluorescence traces (**b**) of ACC-projecting axons of $PL_{VTA–PL}$ neurons in sham and SNI mice. Scale bar, 10 µm.

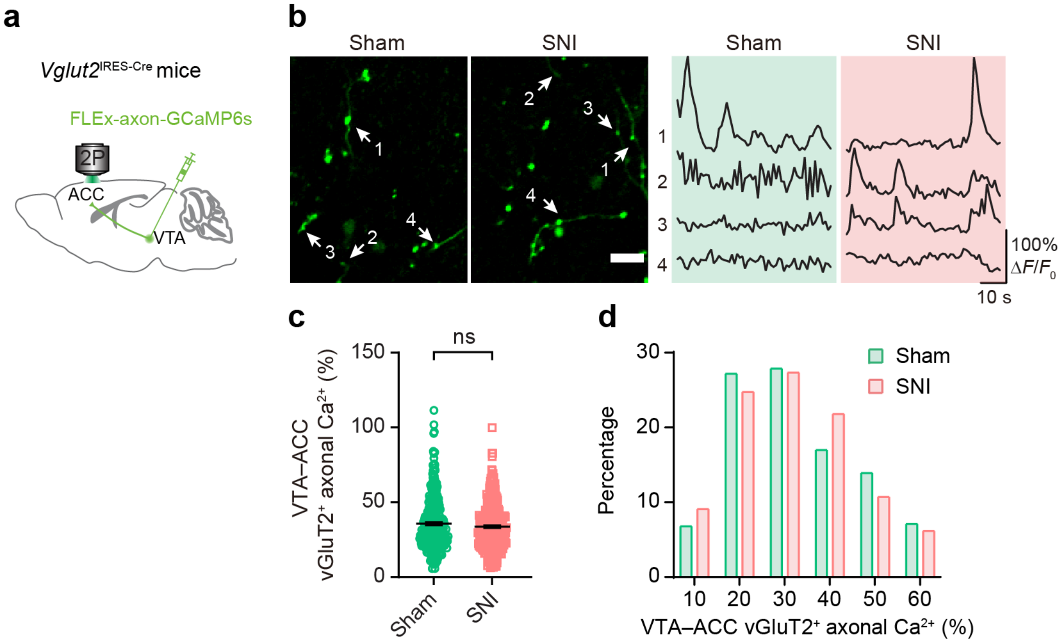

Supplementary Fig. 9: VTA–ACC glutamatergic activity is not altered in mice with neuropathic pain.

a, Experimental design for expressing GCaMP6 in the axons of VTA glutamatergic neurons and performing *in vivo* Ca^{2+} imaging in the ACC two weeks after sham or SNI surgery. **b**, Representative two-photon images and fluorescence traces of ACC-projecting VTA glutamatergic axons expressing GCaMP6. Scale bar, 10 µm. **c**, Ca^{2+} activity in VTA–ACC glutamatergic terminals ($P = 0.3362$; n = 314/5, 319/6 axon segments/mice). **d**, Distribution plot of data shown in **c**. Summary data are presented as mean ± S.E.M. ns, not significant; by two-sided Mann-Whitney test. Source data are provided as a Source Data file.
