# Supplementary material for: A mesocortical glutamatergic pathway modulates neuropathic pain independent of dopamine co-release
Source: Nat Commun. 2024 Jan 20;15:643. doi: 10.1038/s41467-024-45035-2 (PMC10799877; doi:10.1038/s41467-024-45035-2)
Supplement: Supplementary file 3 — Reporting Summary [file 41467_2024_45035_MOESM3_ESM.pdf]

nature portfolio

Corresponding author(s): Guang Yang

Last updated by author(s): Dec 29, 2023

Reporting Summary

Nature Portfolio wishes to improve the reproducibility of the work that we publish. This form provides structure for consistency and transparency in reporting. For further information on Nature Portfolio policies, see our Editorial Policies and the Editorial Policy Checklist.

Statistics

For all statistical analyses, confirm that the following items are present in the figure legend, table legend, main text, or Methods section.

n/a	Confirmed	
☐	☒	The exact sample size (*n*) for each experimental group/condition, given as a discrete number and unit of measurement
☐	☒	A statement on whether measurements were taken from distinct samples or whether the same sample was measured repeatedly
☐	☒	The statistical test(s) used AND whether they are one- or two-sided<br>*Only common tests should be described solely by name; describe more complex techniques in the Methods section.*
☐	☒	A description of all covariates tested
☐	☒	A description of any assumptions or corrections, such as tests of normality and adjustment for multiple comparisons
☐	☒	A full description of the statistical parameters including central tendency (e.g. means) or other basic estimates (e.g. regression coefficient) AND variation (e.g. standard deviation) or associated estimates of uncertainty (e.g. confidence intervals)
☐	☒	For null hypothesis testing, the test statistic (e.g. *F*, *t*, *r*) with confidence intervals, effect sizes, degrees of freedom and *P* value noted<br>*Give P values as exact values whenever suitable.*
☒	☐	For Bayesian analysis, information on the choice of priors and Markov chain Monte Carlo settings
☒	☐	For hierarchical and complex designs, identification of the appropriate level for tests and full reporting of outcomes
☒	☐	Estimates of effect sizes (e.g. Cohen's *d*, Pearson's *r*), indicating how they were calculated

Our web collection on statistics for biologists contains articles on many of the points above.

Software and code

Policy information about availability of computer code

Data collection	Two-photon images were acquired using ScanImage (v5.4). RT-PP and CPP recordings were conducted using ANY-Maze 6.0 software.
Data analysis	Calcium analysis was performed using the NIH ImageJ software (FIJI 1.53s). RT-PP and CPP analyses were conducted using ANY-Maze 6.0 software. Statistical analysis was performed using GraphPad Prism software (v9.0 or v10.1.0).

For manuscripts utilizing custom algorithms or software that are central to the research but not yet described in published literature, software must be made available to editors and reviewers. We strongly encourage code deposition in a community repository (e.g. GitHub). See the Nature Portfolio guidelines for submitting code & software for further information.

Data

Policy information about availability of data

All manuscripts must include a data availability statement. This statement should provide the following information, where applicable:

- Accession codes, unique identifiers, or web links for publicly available datasets
- A description of any restrictions on data availability
- For clinical datasets or third party data, please ensure that the statement adheres to our policy

The data supporting the findings of this study can be found in the paper and its supplementary information. Source data are provided with this paper.

Research involving human participants, their data, or biological material

Policy information about studies with human participants or human data. See also policy information about sex, gender (identity/presentation), and sexual orientation and race, ethnicity and racism.

Reporting on sex and gender	The item is not relevant to this study.
Reporting on race, ethnicity, or other socially relevant groupings	The item is not relevant to this study.
Population characteristics	The item is not relevant to this study.
Recruitment	The item is not relevant to this study.
Ethics oversight	The item is not relevant to this study.

Note that full information on the approval of the study protocol must also be provided in the manuscript.

Field-specific reporting

Please select the one below that is the best fit for your research. If you are not sure, read the appropriate sections before making your selection.

☒ Life sciences ☐ Behavioural & social sciences ☐ Ecological, evolutionary & environmental sciences

For a reference copy of the document with all sections, see nature.com/documents/nr-reporting-summary-flat.pdf

Life sciences study design

All studies must disclose on these points even when the disclosure is negative.

Sample size	No statistical methods were used to pre-determine sample sizes. The sample sizes in this study are consistent with those reported in previous publications(PMIDs: 35561213, 36103825, 36690899). For calcium imaging, 3-10 mice per group were used; behavior tests involved 5-10 mice per group; and circuit dissection studies included 3-8 mice per group.
Data exclusions	No animals that were successfully measured were excluded from the analysis. Imaging segments with excessive movement were removed from data analysis.
Replication	Two-photon imaging was conducted in multiple animals. Behavioral tests were carried out in separate animals to evaluate different treatments. Immunohistochemistry studies were replicated in more than two brain sections per mouse. The results from different animals were consistent.
Randomization	Animals were randomly assigned to treatment groups.
Blinding	The experimenters were blinded to group allocations in all experiments.

Reporting for specific materials, systems and methods

We require information from authors about some types of materials, experimental systems and methods used in many studies. Here, indicate whether each material, system or method listed is relevant to your study. If you are not sure if a list item applies to your research, read the appropriate section before selecting a response.

Materials & experimental systems

n/a	Involved in the study	
☐	☒	Antibodies
☒	☐	Eukaryotic cell lines
☒	☐	Palaeontology and archaeology
☐	☒	Animals and other organisms
☒	☐	Clinical data
☒	☐	Dual use research of concern
☒	☐	Plants

Methods

n/a	Involved in the study	
☒	☐	ChIP-seq
☒	☐	Flow cytometry
☒	☐	MRI-based neuroimaging

Antibodies

Antibodies used	Primary antibodies:Rabbit anti-c-Fos (1:500, 226008; Synaptic Systems), sheep anti-tyrosine hydroxylase (1:800, AB1542; Sigma Aldrich), rabbit anti-HA tag (1:500, ab9110; Abcam), rabbit anti-glutamate (1:500, G6642; Sigma Aldrich).<br>Secondary antibodies: donkey anti-sheep DyLight 405 (1:400, 713475003; Jackson Immuno Research Labs), donkey anti-sheep Alexa Fluor 647 (1:400, A21448; Invitrogen), donkey anti-rabbit CF543 (1:400; 20308, Biotium), donkey anti-rabbit Alexa Fluor 647 (1:400; A31573, Invitrogen).
Validation	Rabbit anti-c-Fos: https://www.sysy.com/product/226008<br>Sheep anti-tyrosine hydroxylase: https://www.sigmaaldrich.com/US/en/product/mm/ab1542<br>Rabbit anti-HA tag: https://www.abcam.com/products/primary-antibodies/ha-tag-antibody-chip-grade-ab9110.html<br>Rabbit anti-glutamate:Tang, HD., et al. Nat Metab 4, 1746–1755 (2022).

Animals and other research organisms

Policy information about studies involving animals; ARRIVE guidelines recommended for reporting animal research, and Sex and Gender in Research

Laboratory animals	Vglut2IRES-Cre (016963), Vglut2IRES-FLPo (030212), Gad2IRES-Cre (010802), PvalbT2A-Cre (012358), SstIRES-Cre (013044), VipIRES-Cre (010908), and C57BL/6J (000664) mice were from the Jackson Laboratory. Thy1.2-GCaMP6s founder line 3 transgenic mice were bred in-house. All mice were group-housed in temperature- and humidity-controlled rooms with a 12-h light-dark cycle. Male and female mice aged two to three months were used for all the experiments.
Wild animals	No wild animals were used in this study.
Reporting on sex	Both male and female mice were used in this study.
Field-collected samples	No field-collected samples were used in this study.
Ethics oversight	All animal procedures were performed in accordance with protocols approved by the Institutional Animal Care and Use Committee at Columbia University as consistent with the National Institutes of Health Guidelines for the Care and Use of Laboratory Animals.

Note that full information on the approval of the study protocol must also be provided in the manuscript.
